# ORAL HEALTH INTERVENTIONS AND TREATMENTS FOR OLDER ADULTS WITH COGNITIVE IMPAIRMENT AND DIABETES

**DOI:** 10.1093/geroni/igad104.1322

**Published:** 2023-12-21

**Authors:** Stephen Shuman, Bei Wu, Michele Saunders

## Abstract

Older adults with cognitive impairment or diabetes have a higher risk of poor oral health. It is therefore critically important to develop effective intervention strategies to improve oral health for these vulnerable populations. In this session, we have included three studies that aim to achieve this goal using different strategies. The first study used stakeholder engaged processes to develop an app that provides information and guidance to caregivers (i.e., paid and family caregivers) to support their oral care provision for older adults with dementia. Findings from this study show that the app was well-accepted and provided an easily accessible tool for caregivers during care provision. The second study aimed to improve oral hygiene outcomes (plaque index and gingival index) for individuals with mild cognitive impairment (MCI) or mild dementia (MD). In this study, the authors identified and compared active behavior change techniques (BCT) used in a care partner-assisted oral health intervention for individuals with MCI and MD using a qualitative, secondary analysis of coaching sessions with care partners. Findings from this study provide insight into the mechanisms of change in individuals’ behaviors using these interventions. The third study was a systematic review aiming to explore the age differences in the effect of multi-component periodontal treatments on oral and metabolic indicators among individuals with periodontitis and diabetes. This study identified 115 eligible trials. This study found that the effect of multi-component periodontal treatments on periodontal outcomes and HbA1c may be more effective among younger populations. This is an Oral Health Interest Group Sponsored Symposium.

